# Anaplastic Carcinoma of the Pancreas Arising from Mucinous Cystic Neoplasm: A Case Report

**DOI:** 10.70352/scrj.cr.26-0405

**Published:** 2026-09-12

**Authors:** Shotaro Horonushi, Masashi Kudo, Ryuhei Noda, Tsuyoshi Terada, Motokazu Sugimoto, Shin Kobayashi, Naoto Gotohda

**Affiliations:** Department of Hepatobiliary and Pancreatic Surgery, National Cancer Center Hospital East, Kashiwa, Chiba, Japan

**Keywords:** anaplastic carcinoma of the pancreas, mucinous cystic neoplasm, mucinous cystic carcinoma, ovarian stroma

## Abstract

**INTRODUCTION:**

Mucinous cystic neoplasm (MCN) is a pancreatic cystic lesion with a generally favorable prognosis; however, malignant transformation can occur. Anaplastic carcinoma of the pancreas is a rare and highly aggressive subtype of pancreatic cancer. Anaplastic transformation arising from MCN is extremely rare, and its clinicopathological features remain unclear. Herein, we report a case in which surgical resection was performed following systemic chemotherapy, highlighting a discrepancy between tumor marker levels and tumor progression.

**CASE PRESENTATION:**

A woman in her 40s presented with abdominal pain. CT revealed a 14-cm multilocular cystic lesion with a solid component in the pancreatic body and tail, accompanied by peritoneal nodules. The tumor marker carbohydrate antigen 19-9 (CA19-9) level was elevated (2100.0 U/mL). The clinical diagnosis was MCN with an associated invasive carcinoma and suspected peritoneal dissemination, and systemic chemotherapy was initiated. Following treatment, the solid component showed progressive enlargement, whereas the CA19-9 level decreased to 33.6 U/mL, and the peritoneal nodules regressed. The tumor subsequently perforated the colon and was complicated by intratumoral infection, requiring percutaneous drainage and laparoscopic ileostomy. Laparoscopic exploration revealed no obvious peritoneal dissemination, and ascitic fluid cytology was negative for malignancy (CY0). Therefore, distal pancreatectomy with partial gastrectomy and partial colectomy was performed. The resected specimen showed a 19-cm solid tumor with scattered multilocular cystic areas. Histopathology revealed highly atypical spindle, pleomorphic, and multinucleated tumor cells in the solid component, while the cystic component was lined by mucinous epithelium exhibiting carcinomatous atypia and associated ovarian-type stroma. The final pathological diagnosis was MCN with an associated invasive carcinoma with pleomorphic-type anaplastic carcinoma. Despite adjuvant chemotherapy, peritoneal recurrence occurred 3 months after surgery, and the patient died 5 months postoperatively.

**CONCLUSIONS:**

Anaplastic carcinoma arising from MCN is extremely rare and exhibits aggressive clinical behavior with a poor prognosis, even after surgical resection. Tumor markers may not accurately reflect disease activity and should be interpreted with caution.

## Abbreviations

CA19-9
: carbohydrate antigen 19-9
CEA
: carcinoembryonic antigen
MCN
: mucinous cystic neoplasm

## INTRODUCTION

MCN is a pancreatic cystic lesion characterized by ovarian-type stroma,^1)^ typically occurring in middle-aged women and arising in the body and tail of the pancreas.^2)^ Although MCN generally has a favorable prognosis, malignant transformation can occur in rare cases.^3,4)^ Anaplastic carcinoma of the pancreas is a rare subtype of invasive ductal carcinoma with an extremely poor prognosis compared with other variants.^5,6)^ Anaplastic transformation arising from MCN is exceedingly rare, and its clinicopathological features and prognosis remain unclear. Herein, we report a case of anaplastic carcinoma arising from MCN that underwent surgical resection following systemic chemotherapy.

## CASE PRESENTATION

**Fig. 1:**
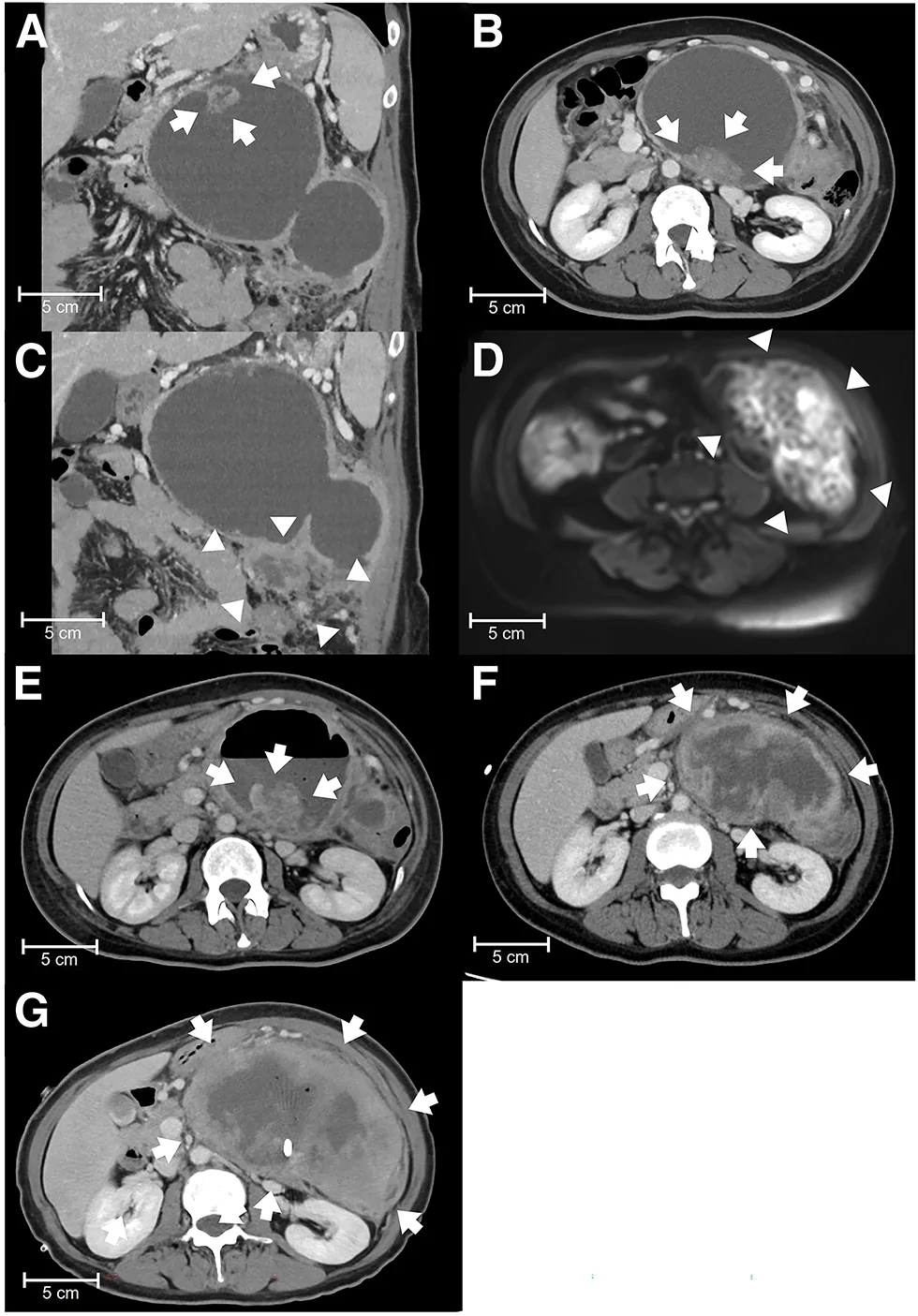
Preoperative imaging. (**A**–**D**) Initial presentation: coronal (**A**) and axial (**B**) CT images showing a 14-cm multilocular cystic lesion in the pancreatic body and tail with an enhancing solid component (arrows). (**C**) Peritoneal nodules around the pancreas (arrowheads). (**D**) MRI showing diffusion restriction in these nodules (arrowheads). (**E**) Three months after the initial diagnosis: CT showing enlargement of the solid component (arrows), tumor perforation into the colon, and intratumoral infection. (**F**) Five months after the initial diagnosis: CT showing enlargement of the solid component to 12.6 cm (arrows). (**G**) Six months after the initial diagnosis: CT showing further enlargement of the solid component to 14.7 cm (arrows).

**Fig. 2:**
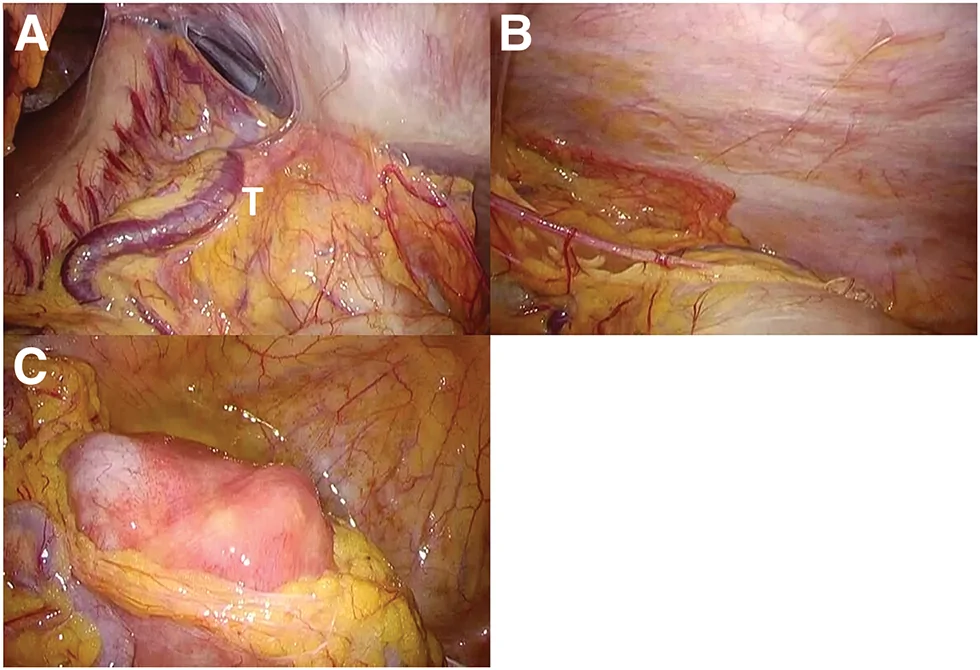
Laparoscopic findings at the time of diverting ileostomy. (**A**) The known tumor (T) in the left upper abdomen showing adhesion to the upper abdominal wall. (**B**) No visible peritoneal nodules were identified in the area where peritoneal dissemination had been suspected on initial imaging studies. (**C**) A small amount of serous ascites was present in the pelvis, and cytological examination of the ascitic fluid was negative for malignancy (CY0).

**Fig. 3:**
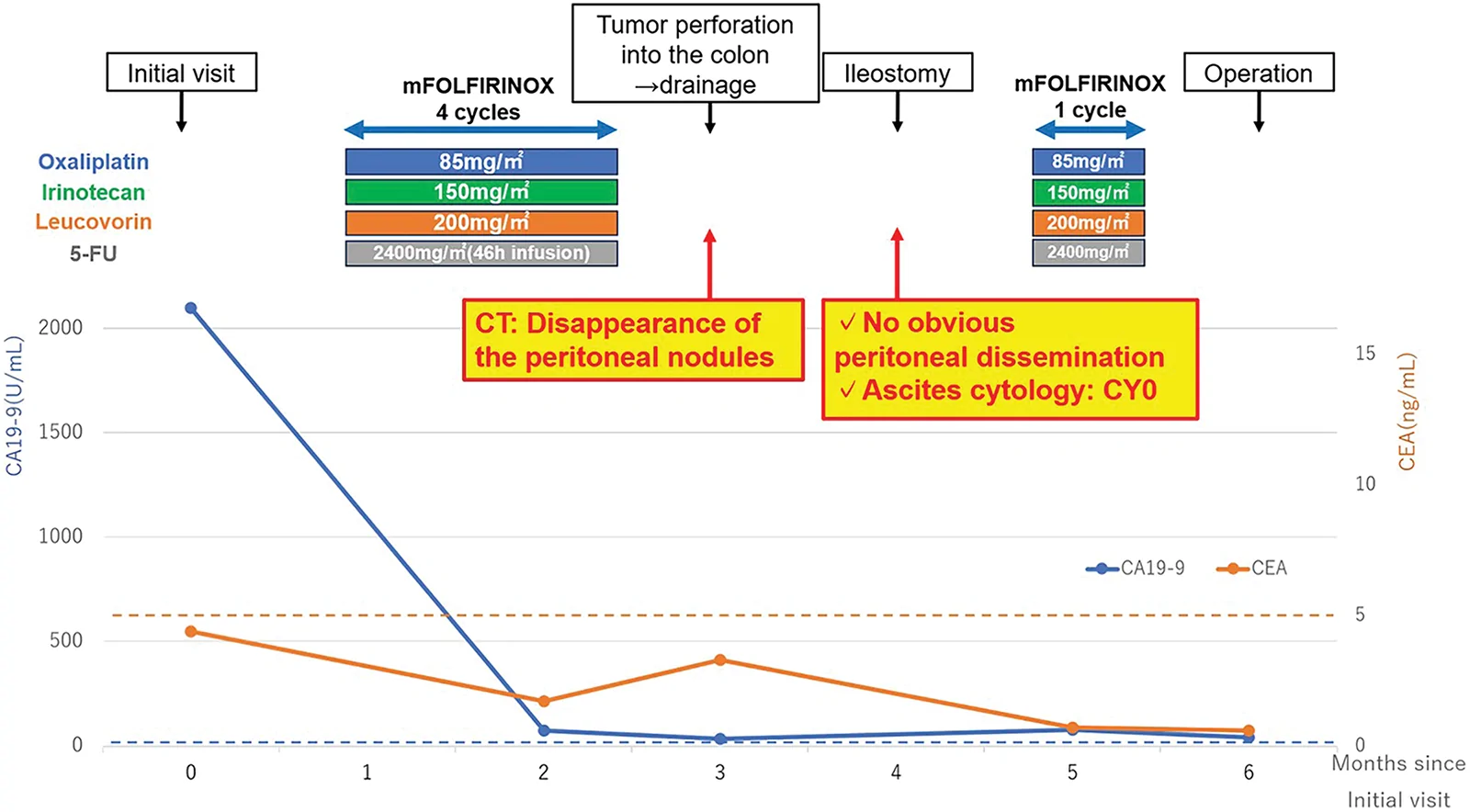
Preoperative clinical course. CA19-9 levels markedly decreased after initiation of mFOLFIRINOX, despite enlargement of the solid tumor component. 5-FU, fluorouracil; CA19-9, carbohydrate antigen 19-9; CEA, carcinoembryonic antigen

**Fig. 4:**
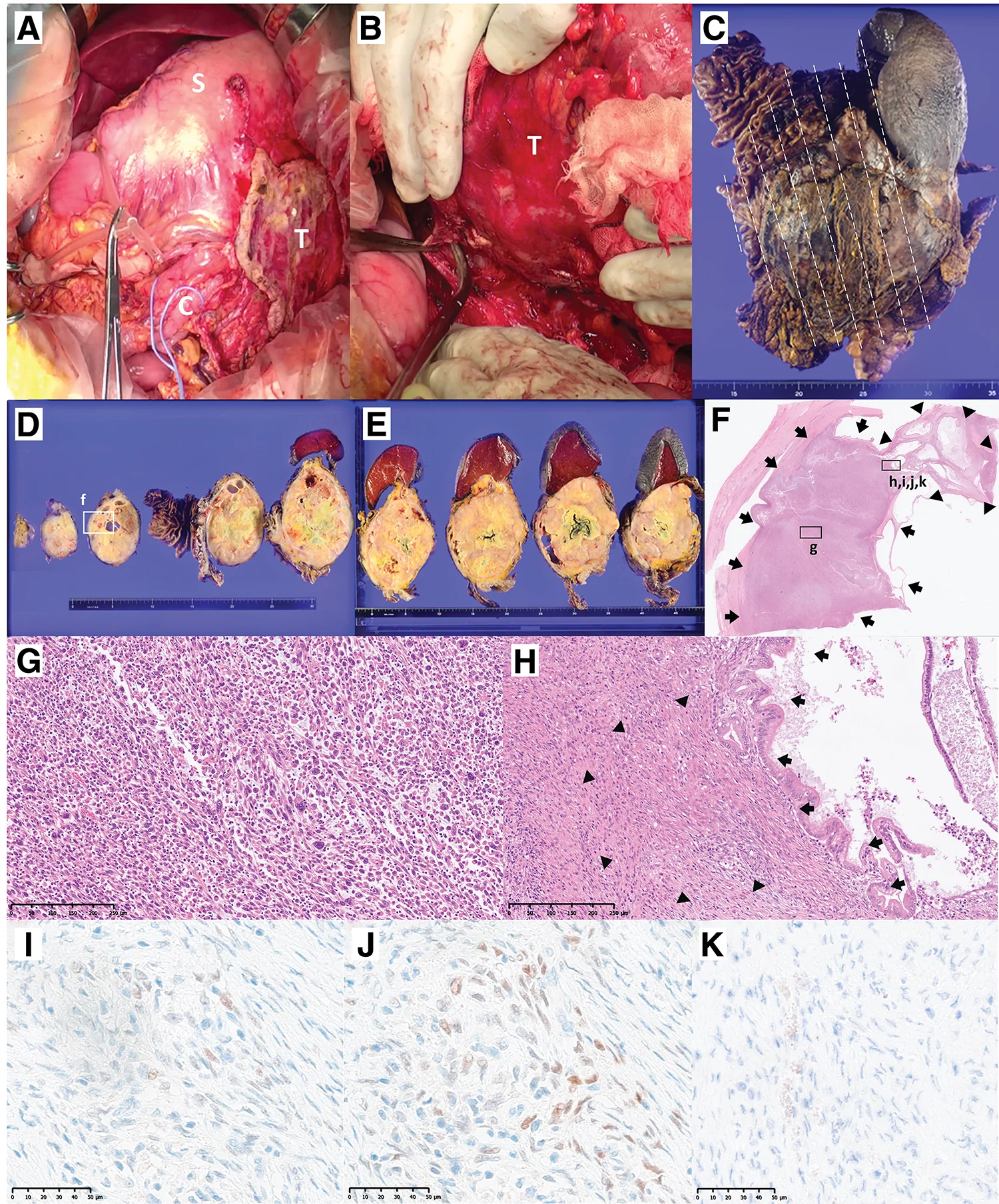
Intraoperative and pathological findings. (**A**) A large cystic tumor (T) was identified in the pancreatic body and tail, invading the stomach (S) and colon (C). (**B**) The tumor (T) was dissected, and distal pancreatectomy with partial gastrectomy and partial colectomy was performed. (**C**–**E**) Macroscopic findings of the resected specimen showed a solid tumor with scattered multilocular cystic areas. (**F**) The tumor consisted of both solid (arrows) and cystic (arrowheads) components (hematoxylin and eosin staining; original magnification, low). (**G**) The solid component consisted of highly atypical spindle, pleomorphic, and multinucleated tumor cells (hematoxylin and eosin staining; original magnification, ×100). (**H**) The cystic component was lined by mucinous epithelium with carcinomatous atypia (arrows) and accompanied by ovarian-type stroma (arrowheads) (hematoxylin and eosin staining; original magnification, ×100). (**I**–**K**) The ovarian-type stroma was positive for estrogen receptor (**I**), progesterone receptor (**J**), and calretinin (**K**) (immunohistochemical staining; original magnification, ×400).

**Fig. 5:**
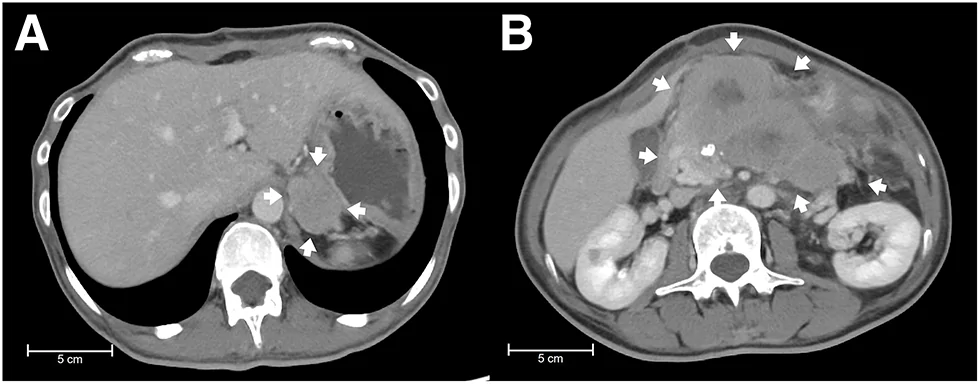
Postoperative imaging. (**A**, **B**) CT revealed peritoneal recurrence 3 months after surgery (arrows).

**Fig. 6:**
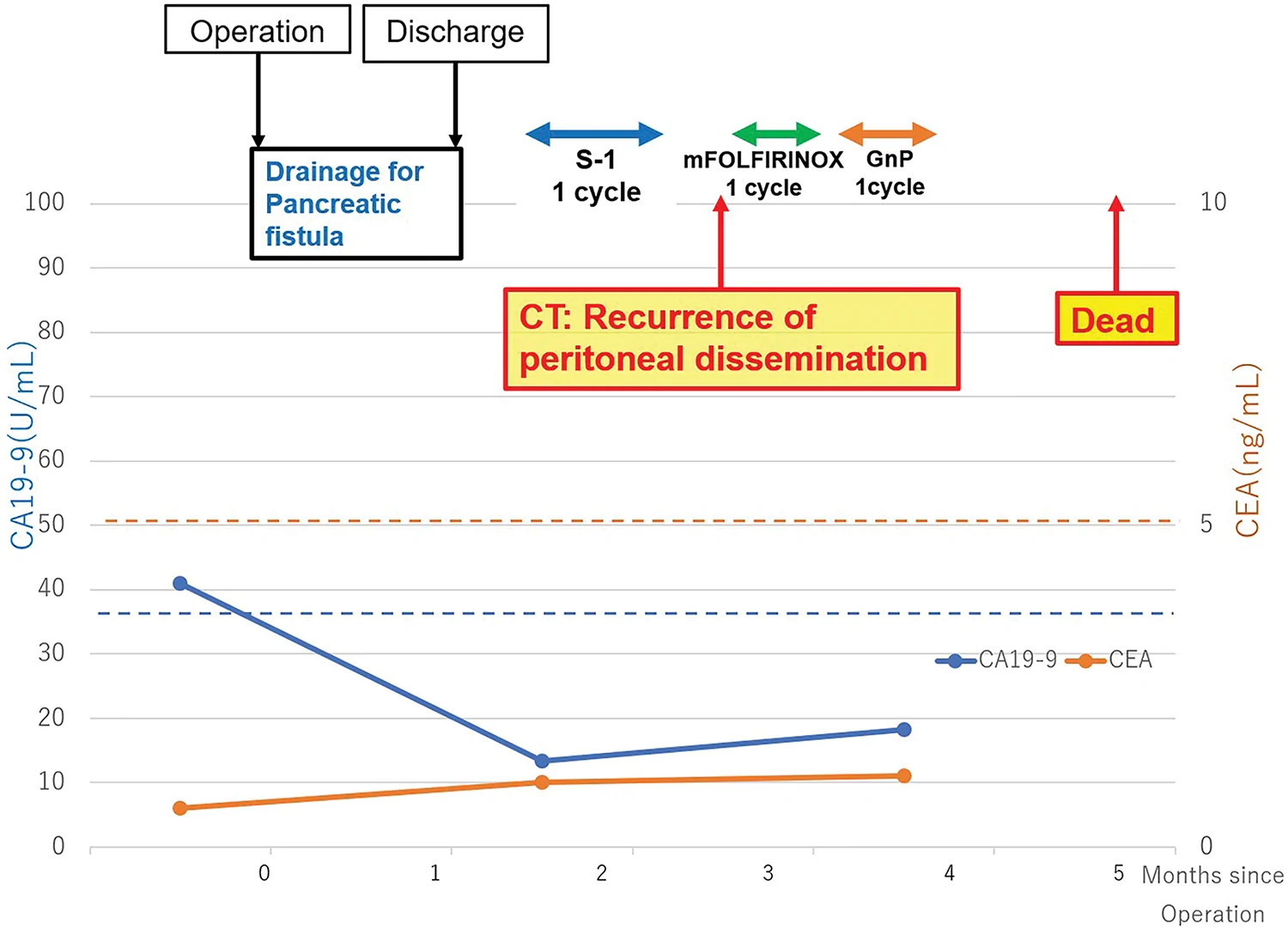
Postoperative clinical course. CA19-9 levels normalized after surgery and remained within the normal range even after recurrence of peritoneal dissemination. CA19-9, Carbohydrate antigen 19-9; CEA, carcinoembryonic antigen; GnP, gemcitabine plus nab-paclitaxel

A woman in her 40s presented with abdominal pain. CT revealed a 14-cm multilocular cystic lesion in the pancreatic body and tail with an enhancing solid component (**Fig. 1A**, **1B**). Peritoneal nodules surrounding the pancreas were also observed (**Fig. 1C**). MRI demonstrated diffusion restriction in these nodules, suggestive of peritoneal dissemination (**Fig. 1D**). The tumor marker CA19-9 level was elevated (2100.0 U/mL). Based on these findings, the clinical diagnosis was MCN with an associated invasive carcinoma and suspected peritoneal dissemination. Therefore, systemic chemotherapy was initiated without pathological confirmation. After 4 cycles of mFOLFIRINOX, the CA19-9 level decreased to 33.6 U/mL, and CT showed disappearance of the peritoneal nodules. However, 3 months after the initial diagnosis, the patient developed worsening abdominal pain. CT revealed enlargement of the solid component, tumor perforation into the splenic flexure of the transverse colon, and intratumoral infection (**Fig. 1E**). Percutaneous abscess drainage and laparoscopic ileostomy were subsequently performed. Laparoscopic exploration revealed the known tumor in the left upper abdomen with adhesion to the upper abdominal wall. No visible peritoneal nodules were identified within the observable abdominal cavity, including the area where peritoneal dissemination had been suspected on the initial imaging studies; therefore, peritoneal biopsy was not performed. A small amount of serous ascites was present in the pelvis, and cytological examination of the ascitic fluid was negative for malignancy (CY0) (**Fig. 2**). Following improvement of her symptoms, one additional cycle of mFOLFIRINOX was administered; however, chemotherapy was discontinued owing to recurrent intratumoral infection accompanied by fever. Although the tumor had increased in size to 14.7 cm (**Fig. 1F**, **1G**), tumor marker levels had decreased, imaging findings showed improvement in the peritoneal nodules, and laparoscopy revealed no evidence of peritoneal dissemination. In addition, continuation of chemotherapy had become difficult because of recurrent intratumoral infection. Therefore, conversion surgery with curative intent and infection control was planned 6 months after the initial diagnosis (**Fig. 3**). A large tumor was identified in the pancreatic body and tail, invading the stomach and colon, and distal pancreatectomy with partial gastrectomy and partial colectomy was performed (**Fig. 4A**, **4B**). The operative time was 487 min, and the blood loss was 1987 mL. Gross examination of the resected specimen showed a 19-cm solid tumor with scattered multilocular cystic areas (**Fig. 4C**–**4E**). Histopathological examination revealed that the solid component, accounting for more than 90% of the tumor, consisted of highly atypical spindle, pleomorphic, and multinucleated tumor cells (**Fig. 4F**, **4G**). In contrast, the cystic component, accounting for less than 10% of the tumor, was lined by mucinous epithelium with carcinomatous atypia and accompanied by ovarian-type stroma that was positive for estrogen receptor, progesterone receptor, and calretinin (**Fig. 4F**, **4H**–**4K**). Tumor invasion into the stomach and colon was observed, with exposure of the tumor on the gastric and colonic mucosal surfaces. Histopathological assessment of the invasion of the splenic artery and vein was difficult. All surgical margins were negative. The final pathological diagnosis was MCN with an associated invasive carcinoma with pleomorphic-type anaplastic carcinoma. According to the Japan Pancreas Society 8th edition classification, the tumor was classified as Pbt, TS4 (190 mm), nodular and cystic type, med, INFa, Ly0, V0, Pn1a, mpd0, ypT3, ypCH0, ypDU0, ypS1, ypRP1, ypPVX, ypAX, ypPLX, ypOO1 (stomach, colon), ypPCM0, ypDPM0, R0, ypN1a (2/17), M0, ypStage IIB. According to the Union for International Cancer Control 9th TNM classification, the tumor was classified as T3N1M0, Stage IIB. The postoperative course was complicated by a pancreatic fistula, which improved with drainage. The patient was discharged 1 month after surgery. Adjuvant chemotherapy with S-1 was initiated; however, peritoneal dissemination recurred 3 months after surgery (**Fig. 5**), whereas CA19-9 levels remained within the normal range (**Fig. 6**). mFOLFIRINOX was subsequently initiated, but tumor progression was observed after a single cycle. The regimen was then changed to gemcitabine plus nab-paclitaxel; however, treatment was discontinued after 1 cycle owing to deterioration of the patient’s general condition. The patient died of the disease 5 months after surgery.

## DISCUSSION

In the present case, the tumor comprised both cystic and solid components. The cystic component showed mucinous epithelium with ovarian-type stroma, which is characteristically positive for estrogen receptor, progesterone receptor, and calretinin,^7,8)^ consistent with our findings. In contrast, the solid component demonstrated pleomorphic-type anaplastic carcinoma. Although no clear transitional zone was identified, the clinical course, characterized by a predominantly cystic lesion at initial presentation followed by rapid growth of the mural nodule, together with the histopathological findings, supported the diagnosis of anaplastic carcinoma arising from MCN. This entity is extremely rare. A PubMed search using the keywords “anaplastic carcinoma,” “mucinous cystic neoplasm,” and “mucinous cystic carcinoma” identified 20 reports comprising 22 cases of anaplastic carcinoma arising from MCN (**Table 1**).^9–28)^ The mean patient age was 47 years, and 20 patients (90.9%) were female. The tumors predominantly occurred in the pancreatic body and tail, with a mean maximum diameter of 12.7 cm. Combined resection of adjacent organs was performed in 6 cases (27.3%). These findings suggest that even when arising from MCN, an anaplastic carcinoma component may confer aggressive local invasiveness. None of the previously reported cases received preoperative chemotherapy, and all underwent upfront surgical resection. Similar to previously reported cases, our patient was a middle-aged woman with a large tumor arising from the pancreatic body and tail. However, to our knowledge, this is the first reported case in which surgical resection was performed after systemic chemotherapy.

**Table 1:** Reported surgically resected cases of anaplastic carcinoma of the pancreas arising from MCN

Year	Authors	Age/Sex	Tumor location	Maximum tumor size (cm)	Surgical procedure	Histological subtype	Preoperative CA19-9 (U/mL)	Recurrence	Site	Time to recurrence	Outcome	Follow-up
1982	Logan^9)^	35/F	Tail	17	DP+gastrectomy	Pleomorphic	ND	Yes	Local	3.5 weeks	Dead	1.5 months
1991	Rego^10)^	45/F	Body and tail	11	DP+colectomy	Pleomorphic	ND	No	–	–	Alive	16 months
1995	Marinho ^11)^	70/F	Body and tail	4.5	DP	ND	ND	ND	–	–	ND	–
1995	Bergman ^12)^	77/F	Head	5	PD	Osteoclast-like giant cell	ND	ND	–	–	ND	–
1997	Lane^13)^	25/F	Tail	15	DP+hepatectomy	Pleomorphic	ND	Yes	Liver	6 months	Alive	6 months
1997	Nishihara ^14)^	52/F	Tail	10	DP	Pleomorphic	655	Yes	Liver	10 months	Dead	19 months
1997	Wenig^15)^	67/M	Tail	19	DP	Spindle cell	ND	Yes	Peritoneum	few months	Dead	15 months
		48/F	Tail	ND	DP	Spindle cell	ND	No	–	–	Alive	12 months
		65/F	Tail	30	DP	Spindle cell	ND	Yes	Peritoneum, pleura	few months	Dead	9 months
2001	Suda^16)^	35/F	Tail	11	DP	Osteoclast-like giant cell	ND	No	–	–	Alive	14 years
2006	Bloomston ^17)^	67/F	Head	4	PD	Pleomorphic	ND	Yes	Liver, peritoneum	few months	Dead	4 months
2007	Pan^18)^	70/F	Body and tail	14	DP	Osteoclast-like giant cell	ND	No	–	–	Alive	4 months
2008	Hirano^19)^	26/F	Body and tail	17	DP	Osteoclast-like giant cell	15	Yes	Local	7 months	Alive	8 months
2008	Hakamada ^20)^	40/F	Tail	14	DP+gastrectomy	Osteoclast-like giant cell	ND	No	–	–	Alive	4 years
2013	Asberry ^21)^	40/F	Tail	8	DP	Pleomorphic	ND	Yes	Local	9 months	Alive	48 months
2016	Aldaoud ^22)^	37/F	Body and tail	16	DP+gastrectomy+colectomy	Pleomorphic	ND	Yes	Peritoneum	5 weeks	Dead	7 months
2016	Munekage ^23)^	25/F	Body and tail	12.5	DP+colectomy	Pleomorphic	ND	Yes	Peritoneum	20 months	Dead	32 months
2017	Paniccia ^24)^	36/F	Tail	10	DP	Pleomorphic	1175	No	–	–	Alive	8 months
2019	Fan^25)^	54/M	Body	6	DP	Osteoclast-like giant cell	30	No	–	–	Alive	6 months
2021	Hisanaga ^26)^	20s/F	Tail	16	DP	Spindle cell	ND	No	–	–	Alive	6 months
2024	Kapatia ^27)^	62/F	Body	16	DP	Osteoclast-like giant cell	ND	ND	–	–	Dead	10 months
2025	Kono^28)^	34/F	Body and tail	11	DP	Osteoclast-like giant cell	19	No	–	–	Alive	6 months
2025	Our Case	40s/F	Body and tail	19	DP+gastrectomy+colectomy	Pleomorphic	2100/33.6*	Yes	Peritoneum	3 months	Dead	5 months

*For the present case, the preoperative CA19-9 values represent the initial and post-chemotherapy values.

CA19-9, carbohydrate antigen 19-9; DP, distal pancreatectomy; F, female; M, male; MCN, mucinous cystic neoplasm; ND, not described in the original report; PD, pancreaticoduodenectomy

The mechanism underlying anaplastic transformation in MCN remains unclear. Kono et al.^28)^ suggested that hormone receptor signaling may contribute to anaplastic transformation in a pregnancy-associated case. In the present case, the patient had delivered a child 9 years before the initial diagnosis; therefore, the involvement of hormone receptor signaling could not be assessed.

Tumor markers may not adequately reflect tumor activity in anaplastic carcinoma arising from MCN. Previous studies have reported that elevations of tumor markers are relatively uncommon in anaplastic carcinoma of the pancreas. Strobel et al.^29)^ reported that among 18 patients with anaplastic carcinoma, elevated CEA and CA19-9 levels were observed in only 19% and 14% of patients, respectively. Similarly, Imaoka et al.^5)^ reported that among 55 patients with unresectable anaplastic carcinoma, the median CA19-9 level was 35.7 U/mL (IQR, 16.4–556.0 U/mL), indicating that many cases did not show significant elevation. Furthermore, among previously reported cases of anaplastic carcinoma arising from MCN, preoperative CA19-9 levels were within the normal range in 3 of 5 evaluable cases.^14,19,24,25,28)^ In the present case, a discrepancy between tumor marker levels and tumor progression was observed. Chemotherapy led to improvement of peritoneal dissemination and normalization of CA19-9 levels, whereas the solid tumor component showed rapid progression. This discordance may be related to the transition from adenocarcinoma to anaplastic carcinoma during tumor progression. One possible explanation is that chemotherapy reduced the adenocarcinoma component, which produces tumor markers, whereas the anaplastic carcinoma component was less responsive to treatment. However, as no pathological assessment was performed before chemotherapy, this explanation remains speculative.

The present case suggests that tumor marker levels may not accurately reflect disease activity in histologically heterogeneous tumors such as anaplastic carcinoma arising from MCN. Molecular alterations, including KRAS and TP53 mutations, have been reported in anaplastic pancreatic carcinoma^5)^; however, no reliable biomarker for monitoring disease activity or treatment response has been established. Therefore, when rapid growth of mural nodules is observed during follow-up of MCN, the possibility of anaplastic transformation should be considered, and evaluation should rely on imaging findings and clinical course rather than tumor marker levels alone.

MCN generally has a favorable prognosis after complete resection; however, the development of anaplastic carcinoma is associated with an extremely poor prognosis. Among 19 cases with reported outcomes, recurrence occurred in 10 cases (52.6%). The most common sites of recurrence were peritoneal dissemination, liver metastasis, and local recurrence. Two patients died within 6 months after surgery, indicating the aggressive nature of this disease. Prognosis may vary according to histological subtype. Among 21 cases with reported subtypes, 8 (38.1%) were of the osteoclast-like giant cell type, and only 1 of these cases developed recurrence. This finding suggests that the osteoclast-like giant cell type may be associated with a more favorable prognosis than other subtypes, consistent with observations in conventional pancreatic anaplastic carcinoma.^30)^ In the present case, the tumor was classified as the pleomorphic type, which was consistent with its aggressive clinical course.

## CONCLUSIONS

We report a rare case of anaplastic carcinoma arising from MCN. Rapid growth of mural nodules during the follow-up of MCN may suggest anaplastic transformation. Tumor marker levels may not accurately reflect tumor activity, and careful evaluation using imaging and clinical findings is essential. Even after complete resection, this disease is associated with a poor prognosis and a high risk of early recurrence, necessitating close postoperative surveillance.

## DECLARATIONS

### Availability of data and materials

All data supporting the findings of this work are available within the article.
